# Supplementary material for: Modelling Skylarks (Alauda arvensis) to Predict Impacts of Changes in Land Management and Policy: Development and Testing of an Agent-Based Model
Source: PLoS One. 2013 Jun 6;8(6):e65803. doi: 10.1371/journal.pone.0065803 (PMC3675089; doi:10.1371/journal.pone.0065803)
Supplement: Supporting Information S4 — The skylark ODdox as a zipped archive. (ZIP) [file pone.0065803.s004.zip › Skylark_ODdox/skylarks__all_8h.html]

ALMaSS Skylark ODdox: skylarks\_all.h File Reference


ALMaSS Skylark ODdox
 2.0


- Main Page
- Related Pages
- Classes
- Files

- File List
- File Members

Classes |
Macros |
Typedefs |
Enumerations

skylarks\_all.h File Reference

|  |  |
| --- | --- |
| Classes | |
| class | AdultSkylark\_struct |
| class | Nestling\_struct |
| class | PreFledgeling\_struct |
| class | skClutch\_struct |
| class | SkQualGrid |
| class | skSpiralResult |
| class | SkTerritories |
| class | skTerritory\_struct |
| class | skTTerritory |
| class | Skylark\_Adult |
| class | Skylark\_Base |
| class | Skylark\_Clutch |
| class | Skylark\_Female |
| class | Skylark\_Male |
| class | Skylark\_Nestling |
| class | Skylark\_Population\_Manager |
| class | Skylark\_PreFledgeling |
| class | Skylark\_struct |
| class | TSkylarkList< T > |

|  |  |
| --- | --- |
| Macros | |
| #define | UNREFERENCED\_PARAMETER(P)   (P) |

|  |  |
| --- | --- |
| Typedefs | |
| typedef TSkylarkList  < Skylark\_Clutch \* > | Skylark\_ClutchList |
| typedef TSkylarkList  < Skylark\_Female \* > | Skylark\_FemaleList |
| typedef TSkylarkList  < Skylark\_Male \* > | Skylark\_MaleList |
| typedef TSkylarkList  < Skylark\_Nestling \* > | Skylark\_NestlingList |
| typedef TSkylarkList  < Skylark\_PreFledgeling \* > | Skylark\_PreFledgelingList |

|  |  |
| --- | --- |
| Enumerations | |
| enum | SkylarkObject {     sob\_Clutch, sob\_Nestling, sob\_PreFledgeling, sob\_Male,     sob\_Female   } |
| enum | TTypesOfSkState {     toss\_Initiation = 0, toss\_Developing, toss\_Hatching, toss\_CDying,     toss\_NDeveloping, toss\_NMaturing, toss\_NDying, toss\_PDeveloping,     toss\_PMaturing, toss\_PDying, toss\_MFlocking, toss\_MFloating,     toss\_MArriving, toss\_MImmigrating, toss\_MEmigrating, toss\_MTempLeavingArea,     toss\_MFindingTerritory, toss\_AttractingAMate, toss\_FollowingMate, toss\_ScaringOffChicks,     toss\_MCaringForYoung, toss\_MDying, toss\_MRehousing, toss\_FFlocking,     toss\_FFloating, toss\_FArriving, toss\_FImmigrating, toss\_FEmigrating,     toss\_FTempLeavingArea, toss\_FFindingTerritory, toss\_BuildingUpResources, toss\_MakingNest,     toss\_PreparingForBreeding, toss\_Laying, toss\_StartingNewBrood, toss\_EggHatching,     toss\_Incubating, toss\_StoppingBreeding, toss\_FCaringForYoung, toss\_FDying,     toss\_GivingUpTerritory, toss\_Destroy   } |

---

## Macro Definition Documentation

|  |  |  |  |  |  |
| --- | --- | --- | --- | --- | --- |
| #define UNREFERENCED\_PARAMETER | ( |  | P | ) | (P) |

Referenced by Skylark\_Male::OnFoodMessage().

---

## Typedef Documentation

typedef TSkylarkList< Skylark\_Clutch \* > Skylark\_ClutchList

typedef TSkylarkList< Skylark\_Female \* > Skylark\_FemaleList

typedef TSkylarkList< Skylark\_Male \* > Skylark\_MaleList

typedef TSkylarkList< Skylark\_Nestling \* > Skylark\_NestlingList

typedef TSkylarkList< Skylark\_PreFledgeling \* > Skylark\_PreFledgelingList

---

## Enumeration Type Documentation

enum SkylarkObject

**Enumerator:**
:   |  |  |
    | --- | --- |
    | *sob\_Clutch* |  |
    | *sob\_Nestling* |  |
    | *sob\_PreFledgeling* |  |
    | *sob\_Male* |  |
    | *sob\_Female* |  |

{

sob\_Clutch,

sob\_Nestling,

sob\_PreFledgeling,

sob\_Male,

sob\_Female

} SkylarkObject;

enum TTypesOfSkState

**Enumerator:**
:   |  |  |
    | --- | --- |
    | *toss\_Initiation* |  |
    | *toss\_Developing* |  |
    | *toss\_Hatching* |  |
    | *toss\_CDying* |  |
    | *toss\_NDeveloping* |  |
    | *toss\_NMaturing* |  |
    | *toss\_NDying* |  |
    | *toss\_PDeveloping* |  |
    | *toss\_PMaturing* |  |
    | *toss\_PDying* |  |
    | *toss\_MFlocking* |  |
    | *toss\_MFloating* |  |
    | *toss\_MArriving* |  |
    | *toss\_MImmigrating* |  |
    | *toss\_MEmigrating* |  |
    | *toss\_MTempLeavingArea* |  |
    | *toss\_MFindingTerritory* |  |
    | *toss\_AttractingAMate* |  |
    | *toss\_FollowingMate* |  |
    | *toss\_ScaringOffChicks* |  |
    | *toss\_MCaringForYoung* |  |
    | *toss\_MDying* |  |
    | *toss\_MRehousing* |  |
    | *toss\_FFlocking* |  |
    | *toss\_FFloating* |  |
    | *toss\_FArriving* |  |
    | *toss\_FImmigrating* |  |
    | *toss\_FEmigrating* |  |
    | *toss\_FTempLeavingArea* |  |
    | *toss\_FFindingTerritory* |  |
    | *toss\_BuildingUpResources* |  |
    | *toss\_MakingNest* |  |
    | *toss\_PreparingForBreeding* |  |
    | *toss\_Laying* |  |
    | *toss\_StartingNewBrood* |  |
    | *toss\_EggHatching* |  |
    | *toss\_Incubating* |  |
    | *toss\_StoppingBreeding* |  |
    | *toss\_FCaringForYoung* |  |
    | *toss\_FDying* |  |
    | *toss\_GivingUpTerritory* |  |
    | *toss\_Destroy* |  |

{

toss\_Initiation = 0,

//Clutch

toss\_Developing, toss\_Hatching, toss\_CDying,

//Nestling

toss\_NDeveloping, toss\_NMaturing, toss\_NDying,

//PreFledgeling

toss\_PDeveloping, toss\_PMaturing, toss\_PDying,

//MaleSK

toss\_MFlocking, toss\_MFloating, toss\_MArriving, toss\_MImmigrating, toss\_MEmigrating, toss\_MTempLeavingArea,

toss\_MFindingTerritory, toss\_AttractingAMate, toss\_FollowingMate, toss\_ScaringOffChicks,

toss\_MCaringForYoung, toss\_MDying, toss\_MRehousing,

//FemaleSK

toss\_FFlocking, toss\_FFloating, toss\_FArriving, toss\_FImmigrating, toss\_FEmigrating, toss\_FTempLeavingArea,

toss\_FFindingTerritory, toss\_BuildingUpResources, toss\_MakingNest, toss\_PreparingForBreeding, toss\_Laying,

toss\_StartingNewBrood, toss\_EggHatching, toss\_Incubating, toss\_StoppingBreeding, toss\_FCaringForYoung, toss\_FDying,

toss\_GivingUpTerritory,

// Destroy object state

toss\_Destroy,

} TTypesOfSkState;


- CJT
- MSVC
- ALMaSS Working Source
- Skylark
- skylarks\_all.h
- Generated on Thu Jan 10 2013 13:15:35 for ALMaSS Skylark ODdox by
   1.8.1.1
